# Age-Specific Radiological Risk of ^222^Rn in Drinking Water Wells Along the Sultandağı Fault Zone (Türkiye): A One-Year Monitoring Study

**DOI:** 10.3390/toxics14080670

**Published:** 2026-07-29

**Authors:** Ayla Sandıkcıoğlu Gümüş

**Affiliations:** Department of Physics, Faculty of Science and Literature, Afyon Kocatepe University, Afyonkarahisar 03200, Türkiye; sandikci@aku.edu.tr

**Keywords:** ^222^Rn, groundwater, drinking water wells, annual effective dose, excess lifetime cancer risk, active fault zone

## Abstract

**Background:** Radon in drinking water is a potential source of ionizing radiation exposure, yet long-term monitoring data from active tectonic regions remain limited. This study investigated ^222^Rn activity concentrations in drinking-water wells along the Sultandağı Fault (Afyonkarahisar, Türkiye) and assessed age-dependent radiological risks. **Methods:** ^222^Rn concentrations were measured at two-week intervals in 15 drinking-water wells between June 2021 and June 2022 using a WG-1001 water–gas separation system and an AB-5R radiation monitor. Annual effective dose (AED) and excess lifetime cancer risk (ELCR) were calculated for infants, children, and adults. **Results:** ^222^Rn concentrations ranged from 0.05 to 26.64 Bq L^−1^, with a mean of 6.12 ± 2.79 Bq L^−1^. Only one well exceeded the USEPA guideline level (11.1 Bq L^−1^), whereas all measurements remained below the WHO reference level (100 Bq L^−1^). Seasonal mean concentrations varied between 5.48 ± 2.53 and 7.08 ± 3.32 Bq L^−1^. Mean AED values were 35.94, 13.17, and 15.63 μSv y^−1^ for infants, children, and adults, respectively. Corresponding ELCR values were 1.26 × 10^−4^, 0.46 × 10^−4^, and 0.55 × 10^−4^. **Conclusions:** The results indicate generally low radiological risk from radon in drinking water within the study area, although elevated values were observed in some wells. Higher age-specific dose and risk estimates were obtained for infants. These findings provide long-term data on groundwater ^222^Rn variability in an active fault zone and support regional radiological risk assessment.

## 1. Introduction

Humans have been exposed to natural radiation originating from long-lived radionuclides present in the Earth’s crust since the formation of the planet. Radiation emitted during the decay of these radionuclides constitutes a significant component of natural radiation exposure. A considerable fraction of natural radioactivity on Earth originates from ^235^U, ^238^U, ^232^Th, and ^40^K. The primary sources of uranium and thorium are rocks and soils that make up the Earth’s crust. These radionuclides and their decay products are distributed at varying concentrations in soil, water, air, rocks, building materials, food, and beverages. Radioactive elements present in rocks and soils continuously interact with water and may dissolve and migrate into aquatic environments, thereby contributing to natural radioactivity levels. Consequently, groundwater is generally expected to contain higher levels of radioactivity than surface water. The Earth’s crust generally contains uranium at concentrations ranging from 0.5 to 5 ppm, corresponding to specific activity concentrations of approximately 6.3–62.5 Bq kg^−1^ [1].

Radon, an important component of natural radioactivity, is produced through the alpha decay of ^226^Ra within the ^238^U decay series. With a half-life of 3.82 days, radon (^222^Rn) is a radioactive noble gas that plays a significant role in various geophysical and environmental processes. Naturally occurring radon isotopes also include ^220^Rn and ^219^Rn, which belong to the ^232^Th and ^235^U decay series, respectively. However, due to their relatively short half-lives and limited environmental significance, ^222^Rn is considered the principal contributor to environmental radon exposure.

Radon is a colorless, odorless, and chemically inert gas and is the 86th element of the periodic table. It can migrate through pore spaces and fracture networks in rocks and soils and subsequently enter the atmosphere and groundwater systems. For this reason, the distribution of radon in soil gas and groundwater has been extensively investigated [2]. The environmental behavior of radon is strongly influenced by geological, hydrogeological, and tectonic conditions. Radon generated within rocks and soils can be transported through pores, fractures, and fault-related discontinuities, facilitating its release into surrounding environments [1,2,3]. Active fault zones, in particular, act as natural conduits for the upward migration of deep crustal gases. Consequently, radon distribution is closely associated with tectonic structures, lithological characteristics, and hydrogeological conditions. Fracture systems developed along active faults may enhance radon transport into groundwater systems, thereby influencing groundwater radon concentrations [1,2]. Because the recoil distance of radon atoms is limited, only those generated near mineral surfaces can enter water, making groundwater radon concentrations highly dependent on local geological conditions [4]. Accordingly, elevated radon concentrations have frequently been reported in water resources located within tectonically active regions [5,6,7,8,9]. Fault zones facilitate radon migration by providing preferential pathways for subsurface fluid circulation [6], resulting in radon concentrations that are often higher than regional background levels [10].

Environmental radon exposure accounts for a substantial fraction of the natural radiation dose received by humans [1,3]. Radon progeny, particularly ^218^Po and ^214^Po, are alpha-emitting radionuclides that contribute significantly to internal radiation doses following inhalation [1]. Inhaled radon and its decay products may accumulate in lung tissue, delivering ionizing radiation and increasing the risk of lung cancer following prolonged exposure [3,11,12]. Furthermore, radon progeny can attach to airborne particles, enhancing their deposition within the respiratory tract [2,13]. Drinking water represents another important pathway of radon exposure. Radon dissolved in groundwater may enter the human body through ingestion, while radon released from water during domestic use may also contribute to inhalation exposure [3,14]. Therefore, radon exposure may occur through both inhalation and ingestion pathways, constituting a significant public health concern.

Epidemiological studies have consistently identified smoking as the leading cause of lung cancer, with radon recognized as the second most important environmental risk factor. The World Health Organization (WHO) classifies radon as a human carcinogen and identifies it as the second leading cause of lung cancer after smoking [1,3]. Depending on national smoking prevalence and average indoor radon levels, approximately 3–14% of all lung cancer cases are estimated to be attributable to radon exposure [3]. Moreover, increases in long-term average radon concentrations have been associated with elevated lung cancer risk, with studies reporting an approximately 10–16% increase in risk for every 100 Bq m^−3^ increase in radon concentration [3,15].

Radon studies have been extensively conducted worldwide across various environmental media. Researchers have investigated radon concentrations in soils [16,17], atmospheric air [18,19], residential environments [20,21], workplaces and schools [22,23], groundwater [24,25,26], well waters [27,28], spa waters [29,30], thermal waters [31,32], and spring waters [33,34]. These investigations continue to provide valuable information on the spatial and temporal variability of radon in different environmental systems. Previous studies indicate that radon distribution may be influenced by geological, hydrogeological, and tectonic characteristics. In particular, variations in radon concentrations have been reported in areas located on or near fault zones, where geological and hydrogeological conditions may contribute to differences in radon occurrence [5,10,35,36,37,38,39,40,41].

Groundwater resources provide a substantial proportion of drinking water supplies, particularly in rural and semi-rural regions. Radon present in these resources may pose a potential public health concern due to exposure through drinking water consumption. Therefore, several international organizations have established guideline and reference values for radon concentrations in drinking water. The United States Environmental Protection Agency (USEPA) has proposed a maximum contaminant level goal of 11.1 Bq L^−1^ for radon in drinking water [42], whereas the World Health Organization (WHO) has established a reference level of 100 Bq L^−1^ for radon concentrations in drinking water [43]. These values highlight the importance of regular monitoring and assessment of radon levels in drinking water sources. In drinking-water radon studies, measured concentrations are generally compared with guideline and reference values recommended by international organizations to evaluate potential health risks. However, a considerable proportion of groundwater radon studies are based on single-time sampling, which may not adequately represent the temporal variability of radon concentrations.

Türkiye is located in an actively deforming tectonic region due to the interaction between the Eurasian, African, and Arabian plates and contains numerous active fault systems [44,45]. In particular, the extensional tectonic regime of Western Anatolia has resulted in the development of extensive fault and fracture systems [46,47]. These structures may contribute to hydrogeological processes affecting groundwater circulation and represent one of the factors that may influence radon distribution in groundwater systems.

The study area is located within the Akşehir–Simav Fault System (ASFS), an active tectonic zone associated with the 2002 Afyon earthquake (Mw 6.2). In this context, the present study aimed to determine ^222^Rn concentrations in well waters used for drinking purposes in Çay district and surrounding villages of Afyonkarahisar, situated within the broader tectonic setting of the Akşehir–Simav Fault System (ASFS), and to assess the potential radiological health risks associated with the ingestion of drinking water from the investigated wells. Annual effective dose (AED) and excess lifetime cancer risk (ELCR) were calculated for different age groups. Measurements were performed at two-week intervals over one year, allowing more representative annual mean ^222^Rn concentrations and exposure estimates to be obtained compared with single-time sampling approaches. Additionally, seasonal variations in ^222^Rn concentrations were evaluated.

## 2. Materials and Methods

### 2.1. Geology of the Study Area

The study area is located within the Akşehir–Simav Fault System (ASFS) and encompasses the town of Çay and its surrounding settlements in Afyonkarahisar Province, western Türkiye. Situated in Central-Western Anatolia, the region lies within Turkey’s first- and second-degree seismic hazard zones, indicating significant tectonic activity and a high earthquake potential. Afyonkarahisar is located in the central sector of the ASFS, one of the principal structural elements defining the extensional neotectonic regime of southwestern Türkiye [48]. Extending from Karaman to Sındırgı (Balıkesir), the fault system is a NE–SW-trending discontinuous oblique-slip normal fault zone approximately 500 km long and 10–30 km wide.

Numerous earthquakes have occurred along the active ASFS, particularly on the Akşehir and Simav segments. Since 1921, many earthquakes with moment magnitudes (M_w_) greater than 4.5 have been recorded, and the system is considered capable of generating earthquakes of up to M_w_ 7.0. Among these events, the M_w_ 6.2 earthquake that occurred between Çay and Sultandağı on 3 February 2002 is particularly notable due to the surface ruptures and structural damage it produced. The tectonic deformation observed along the ASFS is consistent with the extensional tectonic regime and associated normal faulting that characterize western Anatolia [44]. Post-earthquake geological and geophysical investigations revealed the activation of previously unmapped or buried secondary fault segments, indicating that the rupture propagated through a complex and distributed fault network. These findings suggest that the 2002 Sultandağı earthquakes were not confined to the previously recognized principal fault segments but also involved structurally significant secondary fault zones within the region [49]. This evidence highlights the complex deformation pattern of the area and emphasizes the importance of detailed fault mapping and reassessment of regional seismic hazards [49].

**Figure 1:**
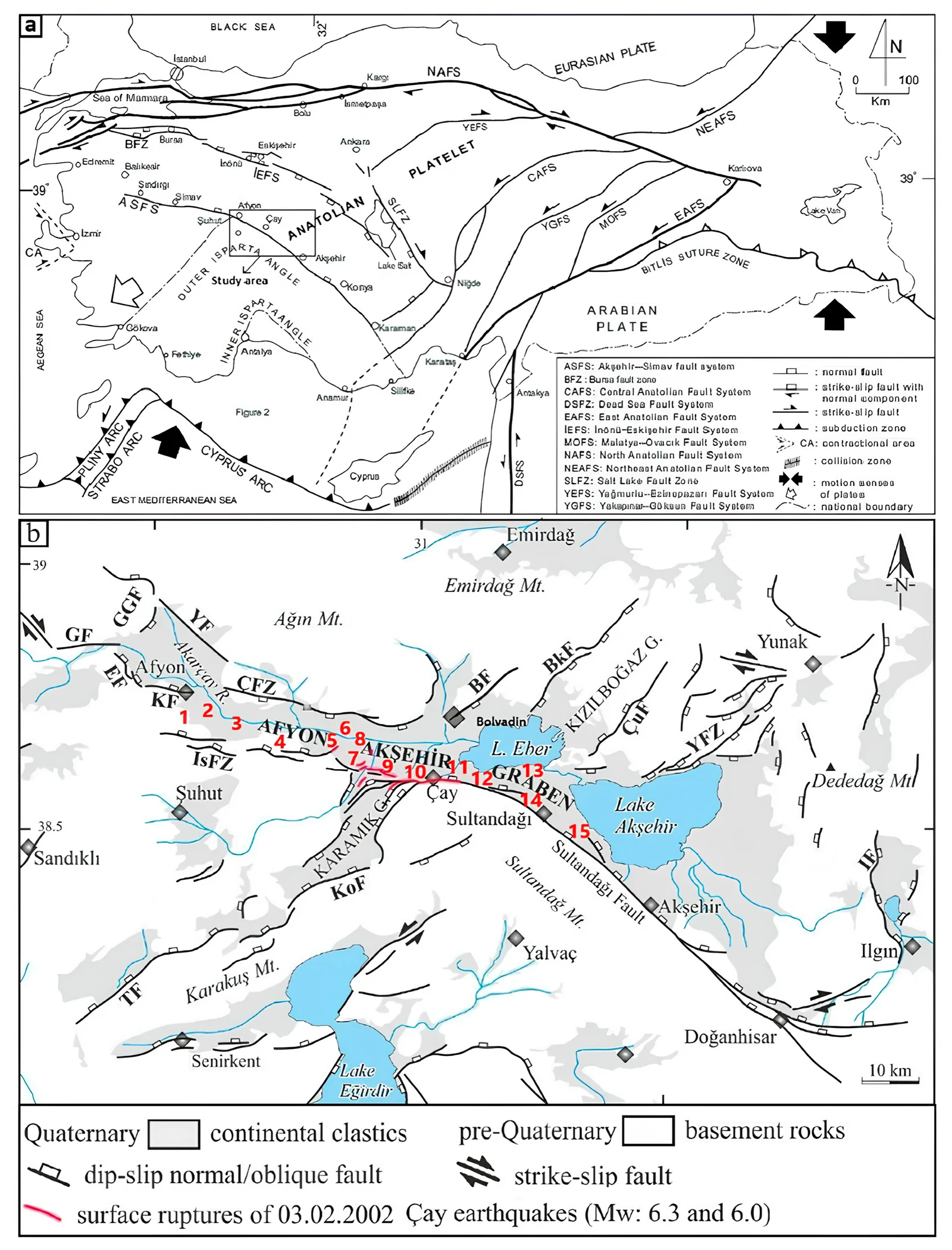
(**a**) Location of the study area and major active fault systems. (**b**) Active fault map of the Afyon–Akşehir Graben (AAG), adapted from [50,51], with sampling locations added. Abbreviations: KG, Karamık Graben; GF, Gecek Fault; GGF, Gazlıgöl Fault; EF, Erkmen Fault; KF, Karahisar Fault; ÇFZ, Çobanlar Fault Zone; IsFZ, Işıklar Fault Zone; YFZ, Yunak Fault Zone; IF, Ilgın Fault; BF, Bolvadin Fault; BkF, Büyük Karabağ Fault; ÇuF, Çukurcak Fault; YF, Yarıkkaya Fault; KoF, Kocbeyli Fault; TF, Tatarlı Fault.

The Akşehir–Simav Fault System comprises numerous active fault segments developed under the influence of the regional extensional tectonic regime. The Sultandağı Fault, located in the southwestern part of the system, constitutes one of the major segments of the ASFS. In addition to controlling the morphotectonic evolution of the region, this structural framework may also influence groundwater circulation and hydrogeological processes. Accordingly, while the regional tectonic setting is discussed within the context of the ASFS, radon measurements were conducted along the Sultandağı Fault. Sampling locations were selected from drinking water wells situated in areas affected by the Sultandağı Fault and associated structural features. The spatial distribution of the sampled wells relative to the Akşehir–Simav Fault System and the Sultandağı Fault is presented in Figure 1.

All investigated groundwater wells are located within Quaternary alluvial deposits of the Afyon–Akşehir Graben. All groundwater samples analyzed in this study were collected from wells located within the same surficial geological unit [50,51].

### 2.2. Sampling and Measurements

#### 2.2.1. Concentration of Radon in Groundwater

Fifteen deep groundwater wells located on or in close proximity to the Sultandağı Fault and used for drinking water supply were selected for this study. Sampling locations were chosen based on their proximity to the Sultandağı Fault and their active use by local residents. In the rural settlements within the study area, groundwater from these wells constitutes an important source of drinking water and is consumed daily by a portion of the local population. Therefore, determining radon levels in these water resources is important from a public health perspective. The groundwater from these wells is consumed directly after extraction without intermediate storage, aeration, treatment, blending, or distribution processes. Therefore, the measured ^222^Rn concentrations were considered representative of the water consumed by the local population. The hydrogeological and physicochemical characteristics of the sampled wells, including well depth, pH, electrical conductivity (EC), and water temperature, are presented in Table 1.

Water samples were collected from the drinking water wells between 19 June 2021 and 14 June 2022 over a 12-month period at approximately two-week (15-day) intervals, and radon concentrations were subsequently determined. For the evaluation of seasonal variation, the measurements were grouped into four meteorological seasons: summer (June–August 2021), autumn (September–November 2021), winter (December 2021–February 2022), and spring (March–May 2022). Seasonal mean ^222^Rn concentrations were calculated for each season. No scheduled sampling campaigns were missed during the monitoring period; therefore, the dataset contained no missing data for the seasonal analysis. Samples were collected directly from the discharge outlet of the operating well pumps into 250 mL borosilicate glass bottles equipped with specially designed airtight caps to prevent gas leakage. Prior to sampling, each well was purged for approximately 5 min to ensure the collection of representative groundwater samples. The bottles were carefully filled to overflowing in order to avoid headspace formation, and the caps were immediately sealed while maintaining continuous overflow to minimize radon loss. All collected samples were transported immediately to the Nuclear Physics Laboratory of Afyon Kocatepe University, where radon analyses were performed on the same day as sample collection.

**Figure 2:**
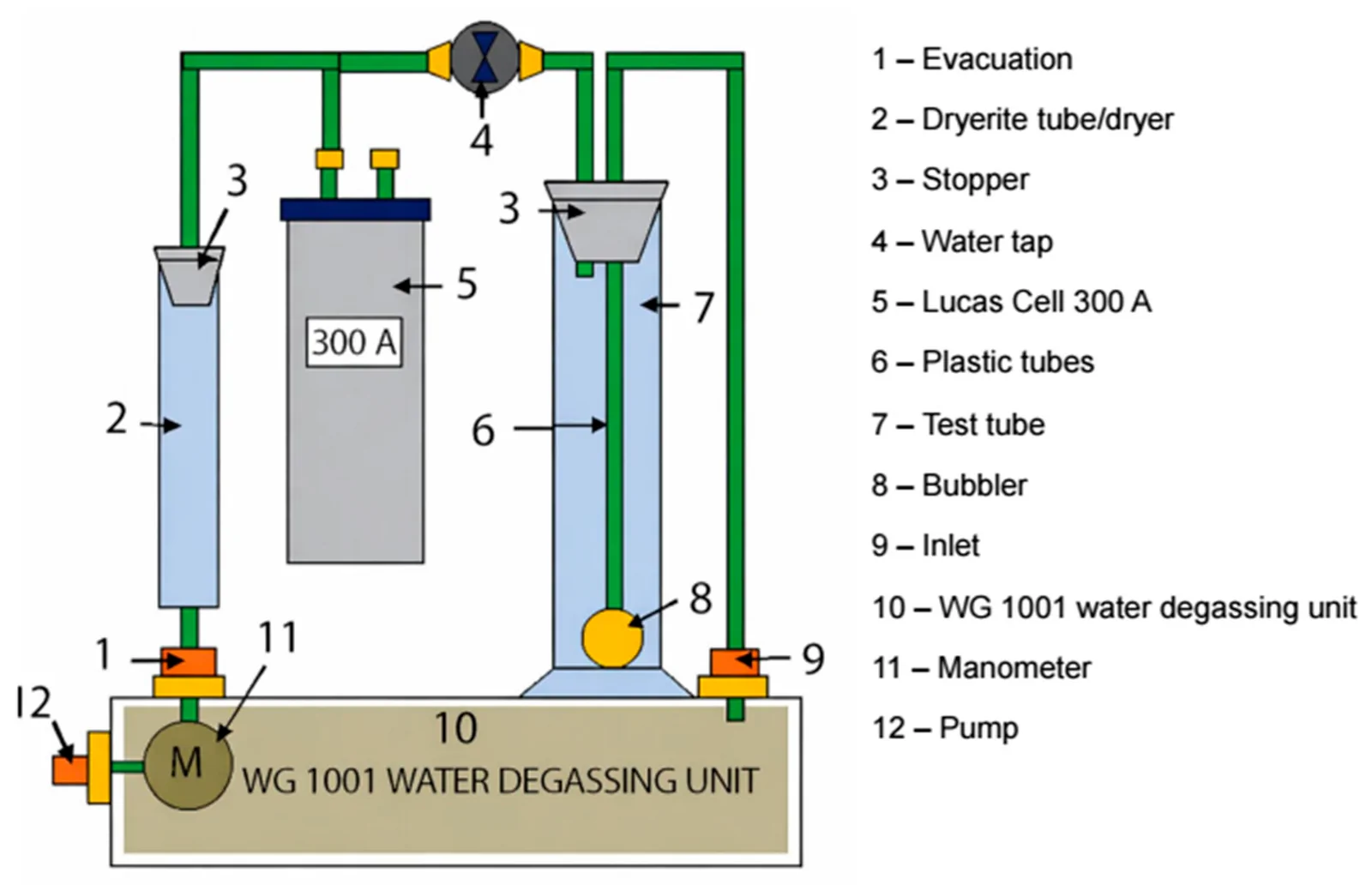
Schematic diagram of the WG-1001 water–gas separation unit and the Lucas scintillation cell-based radon measurement system [53].

**Figure 3:**
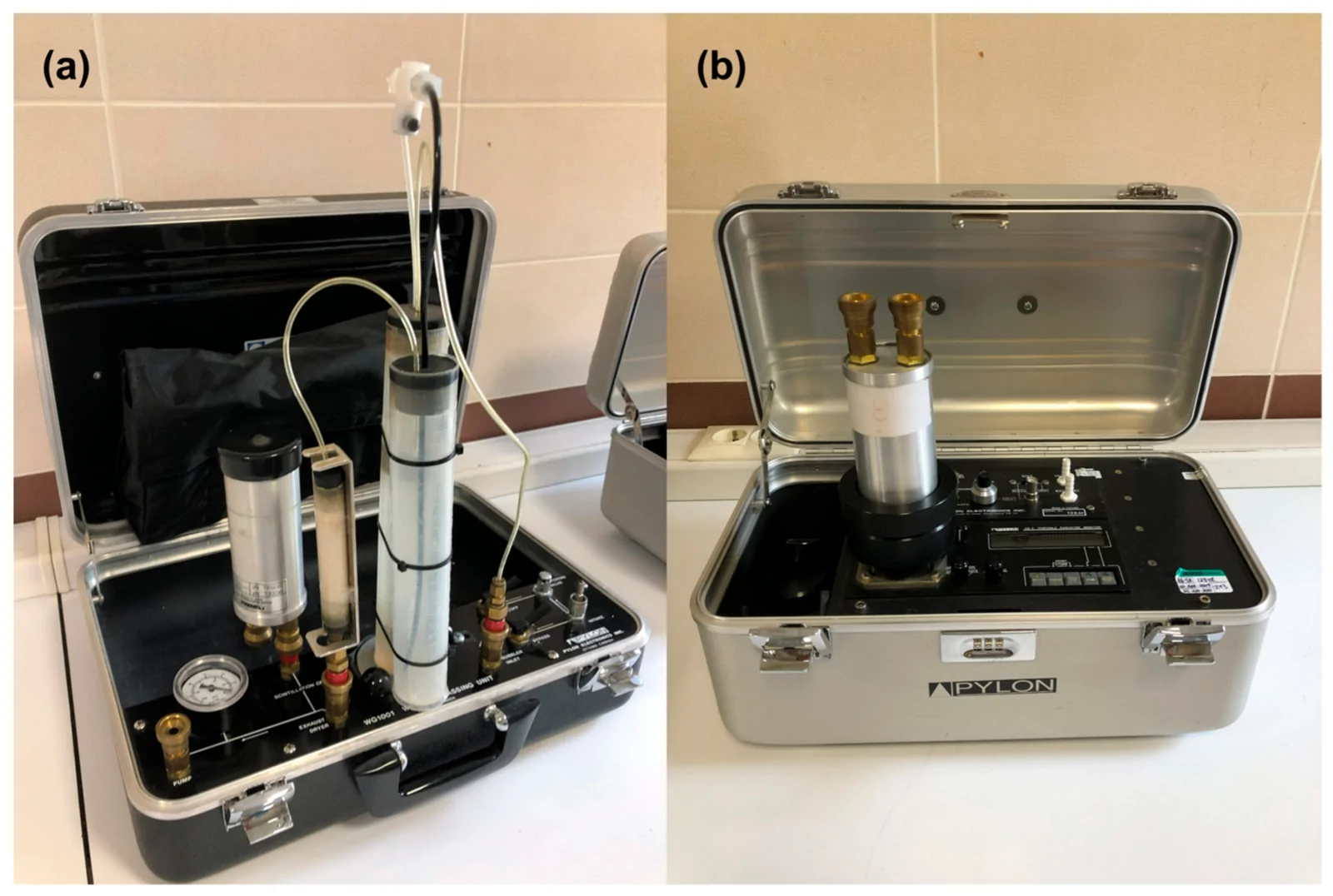
Photographs of the equipment used for ^222^Rn measurements: (**a**) WG-1001 vacuum water–gas separation unit and (**b**) AB-5R radiation monitor.

Radon concentrations in groundwater samples were measured using a WG-1001 vacuum water–gas separation system and an AB-5R radiation monitor manufactured by Pylon Electronics (Pylon Electronic Development Company Ltd., Ottawa, ON, Canada). The AB-5R is a portable radon monitor characterized by high sensitivity and a low background count rate. In this method, radon gas extracted from water by the WG-1001 unit was quantified by counting alpha decays originating from radon and its short-lived progeny using the photomultiplier tube integrated within the Pylon AB-5R portable radon monitor (Pylon Electronic Development Company Ltd., Ottawa, ON, Canada) [52]. Radon measurements were performed using the Lucas scintillation cell technique, which is based on transferring the extracted gas into an evacuated and isolated scintillation chamber for alpha counting. A schematic diagram of the WG-1001 water–gas separation unit and the Lucas scintillation cell-based measurement system is shown in Figure 2. Photographs of the WG-1001 vacuum water–gas separation system and the AB-5R radiation monitor used in this study are presented in Figure 3.

Radon measurements were performed using a leak-tight Pylon LC-300A Lucas-type scintillation cell (Pylon Electronic Development Company Ltd., Ottawa, ON, Canada) internally coated with silver-activated zinc sulfide [ZnS(Ag)]. Following the decay of residual scintillation signals on the ZnS(Ag)-coated surface, the cell was analyzed over three consecutive 5 min counting intervals. The background radiation of each scintillation cell was determined prior to sample analysis by averaging six separate 5 min background measurements (total counting time of 30 min). The resulting values were converted to counts per minute (cpm) and used to determine the background count rate (B) applied in subsequent calculations. A 190 mL water sample was transferred to the degassing vessel connected to the WG-1001 unit. The radon-containing water sample was circulated using the integrated vacuum pump, while the system pressure was continuously monitored with a manometer to ensure appropriate degassing conditions. The system was evacuated to a minimum pressure of 584 mmHg, corresponding to the barometric pressure at an elevation of approximately 1000 m in Afyonkarahisar [52]. Operation under reduced pressure facilitates the transfer of dissolved radon from the aqueous phase to the gas phase, allowing the released radon gas to be drawn into the measurement system through a vacuum line.

To prevent interference during detection and remove moisture, the extracted radon gas was passed through a drying tube filled with desiccant (Drierite). The dried gas was subsequently directed through a bubbler to ensure complete moisture removal before entering the detection chamber. Finally, the gas was transferred into the 300A Lucas scintillation cell. When radon and its short-lived decay products (e.g., ^218^Po) undergo alpha decay, the emitted alpha particles interact with the ZnS(Ag) coating, producing scintillation events. These light pulses are amplified by a photomultiplier tube (PMT), converted into electrical signals, and recorded by the detector system. After the gas extraction process, the Lucas scintillation cell filled with radon gas was stored for 3.5 h to allow radioactive equilibrium to be established between radon and its short-lived progeny. Once equilibrium had been reached, the scintillation cell was placed in the radiation monitor and counted. Six consecutive 5 min counting intervals were performed, yielding a total counting period of 30 min. The mean gross count rate (C) obtained from these six counting intervals was used to calculate the ^222^Rn concentration according to Equation (1), following the manufacturer’s recommended operating procedure. Following the analysis of each water sample, the Lucas scintillation cell was purged according to the manufacturer’s recommended operating procedure to remove residual radon from the previous sample. This cleaning procedure required approximately 20 min and involved repeated evacuation of the cell to approximately 22 inches Hg two to three times, thereby minimizing the influence of residual radon from the previous sample and establishing background conditions [52].

Measured count values were converted to counts per minute (cpm) and defined as the gross count rate (C), while the counting time ($T_{C}$) was recorded. The elapsed time between sample collection $\left(T_{s}\right)$ and counting ($T_{C}$) was also recorded for each sample, and radioactive decay during this interval was corrected using the sample-specific decay correction factor (S) included in Equation (1). Radon concentrations in the water samples were calculated using the equation provided by the manufacturer [52]:

(1) $$A=\frac{\left(C-B\right)\times k}{6.66\times F\times D\times S\times V}$$

where:

*A* is the radon concentration (Bq L^−1^);
*C* is the gross count rate (counts per minute, cpm);
*B* is the background count rate (cpm);
*F* is the counting efficiency of the Lucas cell (0.745 in this study);
*D* is the gas extraction efficiency of the Pylon 300A Lucas cell (0.90);
*S* is the sample-specific decay correction factor selected from the decay correction table provided in the manufacturer’s operating manual according to the elapsed time between the sampling time ($T_{s}$) and counting time ($T_{C}$);
*V* is the sample volume (0.19 L);
*k* is the conversion factor from pCi to Bq (1 pCi = 0.037 Bq);
6.66 is the calibration constant representing the theoretical number of alpha decays per minute produced by 1 pCi of ^222^Rn in equilibrium with its short-lived decay products.

The mean and standard deviation (SD) of ^222^Rn activity concentrations for each well were calculated using data obtained from 27 consecutive measurements conducted throughout the monitoring period. Results are presented as mean ± SD, and minimum and maximum values were additionally reported to characterize the variability of radon concentrations.

#### 2.2.2. Health Risk Assessment

##### Annual Effective Dose from Ingestion of ^222^Rn in Drinking Water

The annual effective dose due to ingestion of ^222^Rn (AED) was calculated for infants, children, and adults using Equation (2) based on the parameters recommended by [1]:

(2) $${\mathrm{AED}}_{\mathrm{ing}}=C_{w}\times W_{L}\times \mathrm{DCF}\times T$$

where **AED_ing_** is the annual effective dose due to radon ingestion from drinking water (Sv y^−1^); **C_w_** is the activity concentration of ^222^Rn in water (Bq L^−1^); **DCF** is the ingestion dose conversion factor for radon [3.5 nSv Bq^−1^ for adults, 5.9 nSv Bq^−1^ for children, and 23 nSv Bq^−1^ for infants], corresponding to the age groups > 17 years (adults), 7–12 years (children), and 1–2 years (infants), respectively [1]; **W**_L_ is the daily water consumption rate [0.75, 1.0, and 2.0 L day^−1^ for infants, children, and adults, respectively]; and **T** is the water consumption period (365 days) [54].

##### Excess Lifetime Cancer Risk (ELCR)

Excess lifetime cancer risk (ELCR) is an indicator of the probability of developing cancer over an individual’s lifetime. The potential lifetime cancer risk associated with long-term exposure to radon in groundwater was evaluated using the ELCR parameter. ELCR calculations are widely employed to assess the potential health effects of radon-contaminated water resources. In this study, ELCR values for the groundwater samples were calculated using Equation (3) [55]:

(3)  ELCR=AED×DL×RF

where **ELCR** is the excess lifetime cancer risk, **AED** is the annual effective dose due to ingestion, **DL** is the average lifespan (70 years), and **RF** is the cancer risk factor (5.5 × 10^−2^ Sv^−1^) [56].

### 2.3. Statistical Analysis

Statistical analyses were conducted using IBM SPSS Statistics for Windows, Version 30.0 (IBM Corp., Armonk, NY, USA) to investigate the relationships between mean ^222^Rn concentrations and selected hydrogeological and physicochemical parameters, including well depth, pH, electrical conductivity (EC), and water temperature. Given the relatively small sample size and the potential non-normal distribution of the dataset, Spearman’s rank correlation coefficient was used to assess the strength and direction of these associations. To evaluate seasonal differences in groundwater ^222^Rn concentrations while accounting for repeated measurements obtained from the same wells, a linear mixed-effects model was applied, with season treated as a fixed effect and well included as a random effect. Regression coefficients (β), 95% confidence intervals (95% CI), and *p*-values were reported. Statistical significance was evaluated at the 95% confidence level, and *p*-values < 0.05 were considered statistically significant.

## 3. Results and Discussion

### 3.1. ^222^Rn Activity Concentrations in Drinking Water Wells

In this study, ^222^Rn activity concentrations measured in drinking water wells located along the Sultandağı Fault were evaluated. Using data collected over the one-year monitoring period, the minimum, maximum, and annual mean radon concentrations were calculated for each well. The measured ^222^Rn concentrations in groundwater samples ranged from 0.05 to 26.64 Bq L^−1^. The lowest concentration was recorded in well W13 during August 2021 (0.05 Bq L^−1^), whereas the highest concentration was observed in well W2 during May 2022 (26.64 Bq L^−1^). The minimum, maximum, and mean radon concentrations for the sampled wells are presented in Table 2.

**Figure 4:**
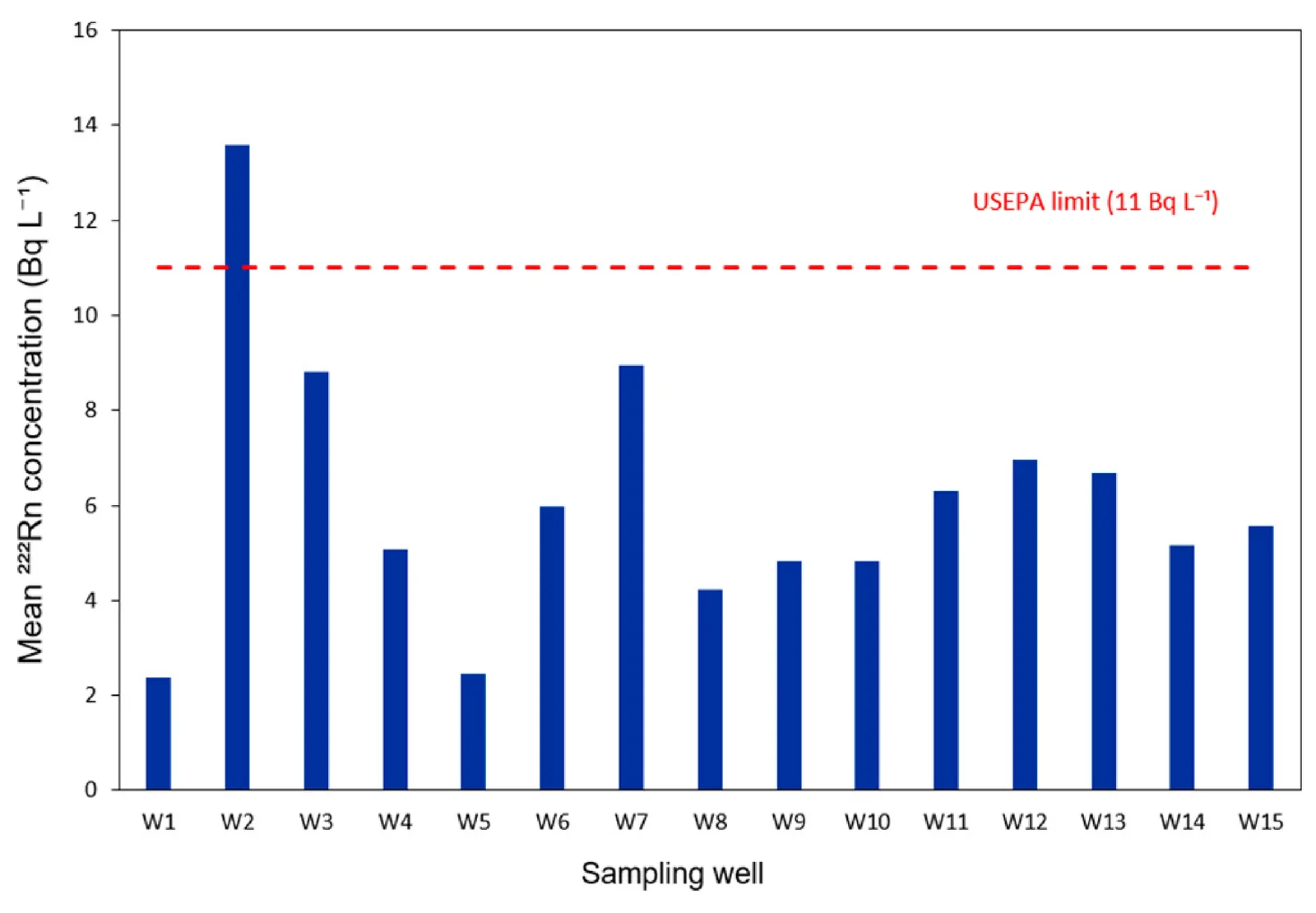
Mean ^222^Rn concentrations measured in the investigated drinking water wells. The red dashed line indicates the USEPA guideline value of 11 Bq L^−1^ [42].

Annual mean ^222^Rn concentrations in the 15 investigated wells ranged from 2.37 to 13.59 Bq L^−1^. The overall mean concentration for the study area was 6.12 ± 2.79 Bq L^−1^, calculated as the arithmetic mean of the annual mean concentrations of all 15 investigated wells. The majority of the wells exhibited mean radon concentrations between 4 and 8 Bq L^−1^ (Figure 4). The highest annual mean radon concentration was observed in well W2 (Salar), with a value of 13.59 Bq L^−1^, whereas the lowest mean concentration was recorded in well W1 (Ali İhsan Paşa), where a value of 2.37 Bq L^−1^ was measured.

As shown in Figure 4, all mean radon concentrations obtained from measurements conducted at two-week intervals over a 12-month period in the drinking water wells remained below the WHO reference level of 100 Bq L^−1^ for radon in drinking water [43]. In addition, the results were evaluated with respect to the USEPA guideline value of 11 Bq L^−1^ [42]. According to this assessment, only the mean radon concentration measured in well W2 (13.59 Bq L^−1^) exceeded the recommended guideline value. Mean radon concentrations in all other wells remained below the USEPA guideline value of 11 Bq L^−1^. These findings indicate that radon concentrations in most of the investigated wells remained below the guideline or reference values for drinking water. However, more detailed monitoring of radon levels may be beneficial at certain locations. It is well known that radon concentrations in groundwater can vary considerably depending on the geological and hydrogeological conditions. These findings are consistent with previous studies investigating radon levels in well waters in Afyonkarahisar. Measurements conducted in ten deep wells located along the Akşehir Fault System reported mean radon concentrations ranging from 2.2 to 7.7 Bq L^−1^ [57]. In the same study, minimum and maximum radon concentrations of 0.7 and 31.7 Bq L^−1^, respectively, were reported. Although different wells were investigated, both studies focused on deep groundwater systems in the same region, allowing regional-scale comparisons. In the present study, mean radon concentrations ranged from 2.37 to 13.59 Bq L^−1^. The lowest mean radon concentrations reported in the two studies were very similar, being 2.2 Bq L^−1^ in [57] and 2.37 Bq L^−1^ in the present study. In contrast, the highest mean radon concentration determined in this study (13.59 Bq L^−1^) was higher than the maximum mean value reported by [57] (7.7 Bq L^−1^). Similarly, the maximum measured radon concentrations were of the same order of magnitude, reaching 31.7 Bq L^−1^ in [57] and 26.64 Bq L^−1^ in the present study.

The wide range of ^222^Rn concentrations reported in groundwater and drinking water worldwide is summarized in Table 3. The mean ^222^Rn concentration determined in the present study (6.12 Bq L^−1^) was higher than the values reported for tap water in Thailand (0.40 Bq L^−1^) [58], groundwater in eastern Saudi Arabia (1.16 Bq L^−1^) [59], and groundwater from the Gandhinagar region of India (2.19 Bq L^−1^) [60]. In contrast, it was substantially lower than the mean radon concentrations reported for hand-dug wells (94.11 Bq L^−1^) and boreholes (82.75 Bq L^−1^) in Dutse, Nigeria [61]. The mean concentration reported for well water in the Peshawar Basin, Pakistan (12.0 Bq L^−1^) [62] was more comparable to the value obtained in the present study.

In the present study, radon concentrations in well waters ranged from 0.05 to 26.64 Bq L^−1^, with a mean value of 6.12 Bq L^−1^. Similarly, a study conducted in Sakarya Province reported radon concentrations ranging from 1.98 to 20.80 Bq L^−1^ in well waters, with a mean concentration of 9.05 Bq L^−1^ [63]. These findings indicate that mean radon concentrations in the Sakarya wells were slightly higher than those observed in the present study. In both studies, the mean radon concentrations remained well below the WHO reference level of 100 Bq L^−1^ for radon in drinking water [43]. Overall, both studies indicate that radon levels in well waters do not pose a significant radiological health risk.

**Table 3:** Comparison of ^222^Rn activity concentrations, annual effective doses (AEDs), and excess lifetime cancer risks (ELCRs) in drinking-water sources reported worldwide and in the present study.

Region/Country	Water Source	^222^Rn Range (Bq L^−1^)	Mean ^222^Rn (Bq L^−1^)	Annual Effective Doses (AEDs) (μSv y^−1^)			Excess Lifetime Cancer Risk (ELCR) (×10^−4^)			Ref.
				Infants	Children	Adults	Infants	Children	Adults	
Dutse, Nigeria	Bore.	31.66–277	82.75	1057.1	906.1	604.1	37.0	31.71	21.14	[61]
Dutse, Nigeria	HDW	33.15–256	94.11	1202.2	1030.5	687.0	42.10	36.10	23.70	[61]
Iluagba, Nigeria	GW/SW	10.6–43.1	24.1	32.3	27.4	17.6	3.17	3.01	2.71	[64]
Ratchathani, Thailand	TW	0.00–2.1	0.40	2.1	0.8	1.0	0.091	0.034	0.044	[58]
Minna, Nigeria	OW	2.1–27.9	10.2	53.2	71.6	74.6	NR	NR	1.0	[65]
Minna, Nigeria	Bore.	2.8–39.2	14.3	74.3	100.0	104.2	NR	NR	1.3	[65]
Peshawar Pakistan	Well	2.6–23.0	12.0	48.0	36.0	35.0	NR	NR	0.84	[62]
SW Punjab, India	SBW	0.89–7.7	3.47	NR	NR	9.0	NR	NR	2.49	[66]
SW Punjab, India	DBW	1.39–11.8	3.97	NR	NR	9.8	NR	NR	2.74	[66]
Kerala, India	GW	0.64–79.9	8.13	4.1	1.5	2.0	NR	NR	0.80	[67]
Gandhinagar, India	GW	0.07–8.3	2.19	7.5	4.5	3.8	NR	NR	NR	[68]
Eastern Saudi Arabia	GW	0.03–3.2	1.16	6.1	2.3	2.9	NR	NR	NR	[59]
Ekiti, Nigeria	HDW	0.6–36.2	13.3	NR	NR	34.1	NR	NR	NR	[69]
Ekiti, Nigeria	HPB	7.0–41.5	23.3	NR	NR	59.6	NR	NR	NR	[69]
Ekiti, Nigeria	MB	0.6–27.4	7.4	NR	NR	19.0	NR	NR	NR	[69]
Kahramanmaraş, Türkiye	Bottled	0.0135–0.0317	0.0212	0.074	0.044	0.054	NR	NR	0.0024	[70]
Sakarya, Türkiye	Well	1.98–20.8	9.05	27.5	16.4	13.9	NR	NR	NR	[63]
Çorlu, Türkiye	Well	22.04–63.92	43.25	NR	NR	8.7	NR	NR	NR	[33]
Afyonkarahisar, Türkiye	Well	0.42–28.8	NR	NR	NR	73.8	NR	NR	NR	[57]
This study	Well	0.05–26.64	6.12	35.9	13.2	15.6	1.26	0.46	0.55	

Abbreviations: Bore., bore; HDW, hand-dug well; OW, open well; HPB, hand-pumped borehole; MB, motorized borehole; SBW, shallow borewell; DBW, deep borewell; GW, groundwater; SW, surface water; TW, tap water; GW/SW, mixed groundwater and surface water; AED, annual effective dose; ELCR, excess lifetime cancer risk; NR, not reported. Note: Values reported for the present study represent arithmetic mean values calculated from annual average ^222^Rn concentrations measured in 15 drinking water wells over a one-year monitoring period. The studies summarized in this table were conducted under diverse geological and hydrogeological conditions, which strongly influence groundwater ^222^Rn concentrations. Accordingly, the reported values are intended to provide a general comparison, and site-specific geological and hydrogeological differences should be considered when interpreting the results. It should be noted that differences in assumptions used for dose and risk calculations, including water consumption rates, dose conversion factors, and exposure durations, may contribute to the variability among reported risk estimates in previous studies. Accordingly, comparisons based on radon concentrations provide a more consistent basis for evaluation, whereas dose and risk values should be interpreted considering the methodological differences among studies.

When compared with previous radon studies conducted in groundwater and drinking water resources in Türkiye, the mean ^222^Rn concentration determined in the present study (6.12 ± 2.79 Bq L^−1^) was considerably lower than the value reported for well waters in the Çorlu region (43.25 ± 7.92 Bq L^−1^) [33]. Similarly, the mean annual effective dose reported in the Çorlu study was 8.65 ± 1.58 μSv y^−1^.

The mean ^222^Rn concentration determined in the present study (6.12 ± 2.79 Bq L^−1^) was substantially higher than that reported for commercially bottled drinking waters in Kahramanmaraş (0.021 ± 0.002 Bq L^−1^) [70]. Likewise, the maximum concentration measured in this study (26.64 Bq L^−1^) greatly exceeded the maximum value reported in the bottled-water study (0.0317 Bq L^−1^). In bottled waters, radioactive decay during storage and transportation, together with radon degassing from water into the atmosphere during shelf life, can substantially reduce radon concentrations in bottled waters. The potential effects of selected hydrogeological parameters on groundwater ^222^Rn concentrations were also evaluated. For this purpose, Spearman correlation analysis was performed using the mean ^222^Rn concentrations and measured physicochemical parameters of the investigated wells. The results are presented in Table 4.

**Figure 5:**
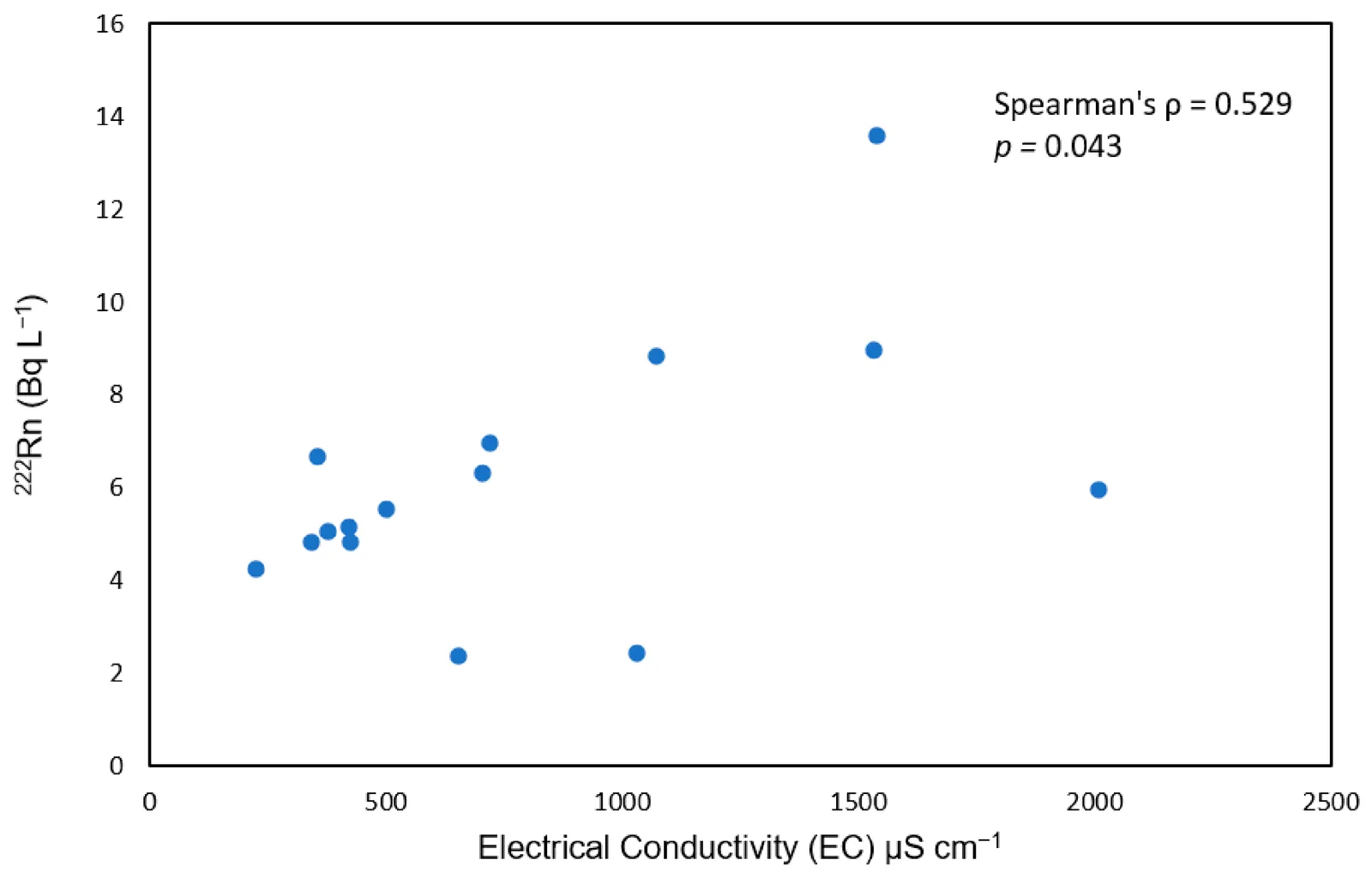
Relationship between mean ^222^Rn concentration and electrical conductivity (EC) in the investigated groundwater wells. Spearman rank correlation analysis indicated a moderate positive correlation (ρ = 0.529, *p* = 0.043).

As electrical conductivity (EC) was the only parameter significantly correlated with ^222^Rn concentrations, the relationship between these variables is illustrated in greater detail in Figure 5.

The correlation analysis revealed no statistically significant relationships between ^222^Rn concentrations and well depth, pH, or water temperature (*p* > 0.05). This finding suggests that well depth, pH, and water temperature alone do not adequately explain the observed variability in groundwater ^222^Rn concentrations in the study area. However, a moderate and statistically significant positive correlation was identified between ^222^Rn concentration and electrical conductivity (EC) (ρ = 0.529, *p* = 0.043).

As EC is an indicator of dissolved ion content, the observed positive relationship may be associated with water–rock interaction processes and the resulting degree of mineralization. Similar positive relationships between groundwater radon concentrations and EC have also been reported by [71,72]. Ref. [71] reported a strong correlation between groundwater ^222^Rn concentrations and EC, whereas [72] described this relationship as a moderate positive correlation (r = 0.39). The moderate positive correlation observed in the present study (ρ = 0.529) is consistent with these previous findings. This suggests that EC may serve not as a parameter directly controlling groundwater ^222^Rn concentrations, but rather as an indicator of hydrogeochemical processes such as water–rock interaction and dissolved ion content. However, given the moderate strength of the correlation and the limited number of well-level observations, the observed relationship should not be interpreted as evidence of a direct cause-and-effect relationship. Nevertheless, the relationship between EC and ^222^Rn appears to vary depending on the hydrogeological setting. Ref. [73] found no significant correlation between groundwater ^222^Rn concentrations and EC and concluded that radon distribution is primarily controlled by rock characteristics, ^226^Ra content, emanation coefficient, and structural features. Similarly, previous studies have emphasized that high radon production potential or high emanation capacity does not necessarily result in elevated groundwater radon concentrations, as radon transport and accumulation in groundwater are also influenced by hydrogeological processes such as fracture systems, groundwater circulation, and water–rock interactions [73,74]. Spearman correlation analysis revealed no statistically significant relationship between mean ^222^Rn concentrations and the distance to the nearest mapped fault (ρ = 0.361, *p* = 0.187).

The annual effective doses (AEDs) due to ingestion, calculated from the measured ^222^Rn concentrations in groundwater, are presented in Table 2. The calculated AED values ranged from 13.90 to 79.86 μSv y^−1^ for infants, 5.09 to 29.27 μSv y^−1^ for children, and 6.04 to 34.72 μSv y^−1^ for adults. The corresponding mean AED values were 35.94, 13.17, and 15.63 μSv y^−1^ for infants, children, and adults, respectively. At all sampling locations, the highest AED values were obtained for infants, followed by adults and children. This difference may be attributed to age-dependent dose conversion factors and differences in daily water consumption rates used in the dose calculations.

**Figure 6:**
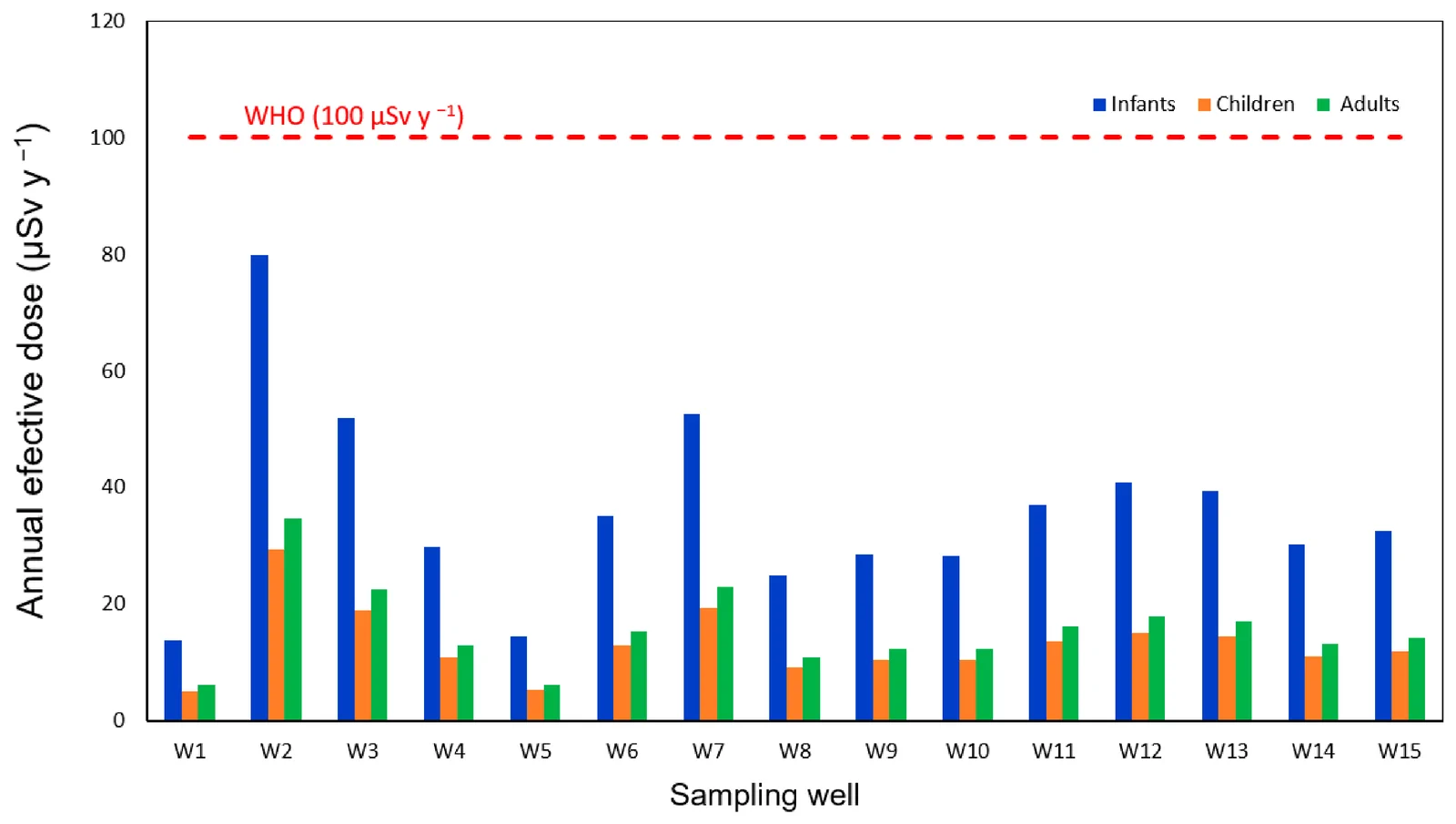
Annual effective dose from ingestion of groundwater for infants, children and adults at the sampled wells (W1–W15). The red dashed line indicates the WHO guideline value of 100 µSv y^−1^ [54].

Among the investigated wells, the highest AED values were observed in well W2, whereas the lowest values were calculated for well W1. The variation in annual effective doses primarily reflects the differences in measured radon concentrations among groundwater samples. However, as shown in Figure 6, even the highest AED value (79.86 μSv y^−1^ for infants in well W2) remained below the WHO reference dose criterion of 100 μSv y^−1^, recommended for drinking water quality [54]. Furthermore, all calculated AED values were lower than the reference dose level of 290 μSv y^−1^ recommended by [1].

The obtained results indicate that, under the current conditions, the groundwater resources in the study area do not pose a significant radiological risk through drinking-water consumption. Nevertheless, the relatively higher AED values observed in some wells highlight the importance of regular monitoring of radon levels in groundwater. When compared with the literature data summarized in Table 3, the AED and ELCR values calculated in this study were lower than those reported for many regions and comparable to those reported in some previous studies. However, it should be noted that differences in AED and ELCR values among studies are not solely attributable to variations in radon concentrations, as dose conversion factors, age-group classifications, water-consumption rates, and risk coefficients may differ between studies.

The excess lifetime cancer risk (ELCR) values associated with ^222^Rn concentrations in groundwater are presented in Table 2. The calculated ELCR values ranged from 0.49 × 10^−4^ to 2.80 × 10^−4^ for infants, from 0.18 × 10^−4^ to 1.02 × 10^−4^ for children, and from 0.21 × 10^−4^ to 1.22 × 10^−4^ for adults. The mean ELCR values were calculated as 1.26 × 10^−4^, 0.46 × 10^−4^, and 0.55 × 10^−4^ for infants, children, and adults, respectively. At all sampling locations, the highest ELCR values were observed for infants, followed by adults and children. This difference among age groups can be attributed to variations in age-dependent dose conversion factors and daily water-consumption rates used in the calculations.

Among the investigated wells, the highest ELCR values were obtained for well W2, whereas the lowest values were observed for well W1. The variation in ELCR values is directly related to the measured ^222^Rn concentrations in groundwater and the corresponding annual effective doses. The reference risk level of 1 × 10^−4^ was adopted based on the recommendations of the United States Environmental Protection Agency (USEPA), which generally uses a lifetime excess cancer risk range of 10^−6^–10^−4^ for risk management decisions [75]. Comparison with this reference level showed that some of the calculated ELCR values were higher than 1 × 10^−4^ [76]. In particular, the highest ELCR values were recorded in well W2, reaching 2.80 × 10^−4^ for infants, 1.02 × 10^−4^ for children, and 1.22 × 10^−4^ for adults. These findings indicate that wells with elevated radon concentrations may also exhibit higher ELCR values. Therefore, periodic monitoring is recommended, particularly for wells with comparatively high radon levels. Nevertheless, the mean ELCR values for children and adults remained below the reference level of 1 × 10^−4^, and elevated risk values were observed only in a limited number of wells [76]. Furthermore, all measured radon concentrations in the study area remained below the reference level of 100 Bq L^−1^ recommended by the World Health Organization (WHO) for drinking water [43]. These results suggest that the investigated groundwater sources generally pose a low radiological risk through drinking-water consumption, although regular monitoring of radon levels may be beneficial at certain sampling locations.

The age-specific ELCR values presented in this study were derived from age-specific annual effective doses calculated using age-dependent dose conversion factors and water-consumption rates. In the ELCR calculation, the 70-year duration represents the conventional reference lifetime widely adopted in radiological risk assessment rather than the duration of a specific age category [58]. Accordingly, the calculated age-specific ELCR values provide a comparative assessment of radiological risk among different age groups within the adopted assessment methodology. Considering that ^222^Rn concentrations in groundwater may exhibit temporal variability, health-risk assessments based on long-term monitoring data are likely to provide more reliable estimates than those based on single measurements.

Measurements conducted at approximately two-week intervals over a one-year period revealed that some wells exhibited consistently higher ^222^Rn concentrations than others. Previous studies have reported that significant differences in ^222^Rn concentrations may occur even among wells located within the same geological unit and in close proximity to one another [73]. Such differences have been attributed to variations in geological and hydrogeological characteristics of aquifers, groundwater-flow conditions, and groundwater residence times [73,77]. In addition, temporal and seasonal variations in groundwater ^222^Rn concentrations have been reported in several studies [78,79].

### 3.2. Seasonal Variation of ^222^Rn Activity Concentrations in Drinking Water Wells

**Figure 7:**
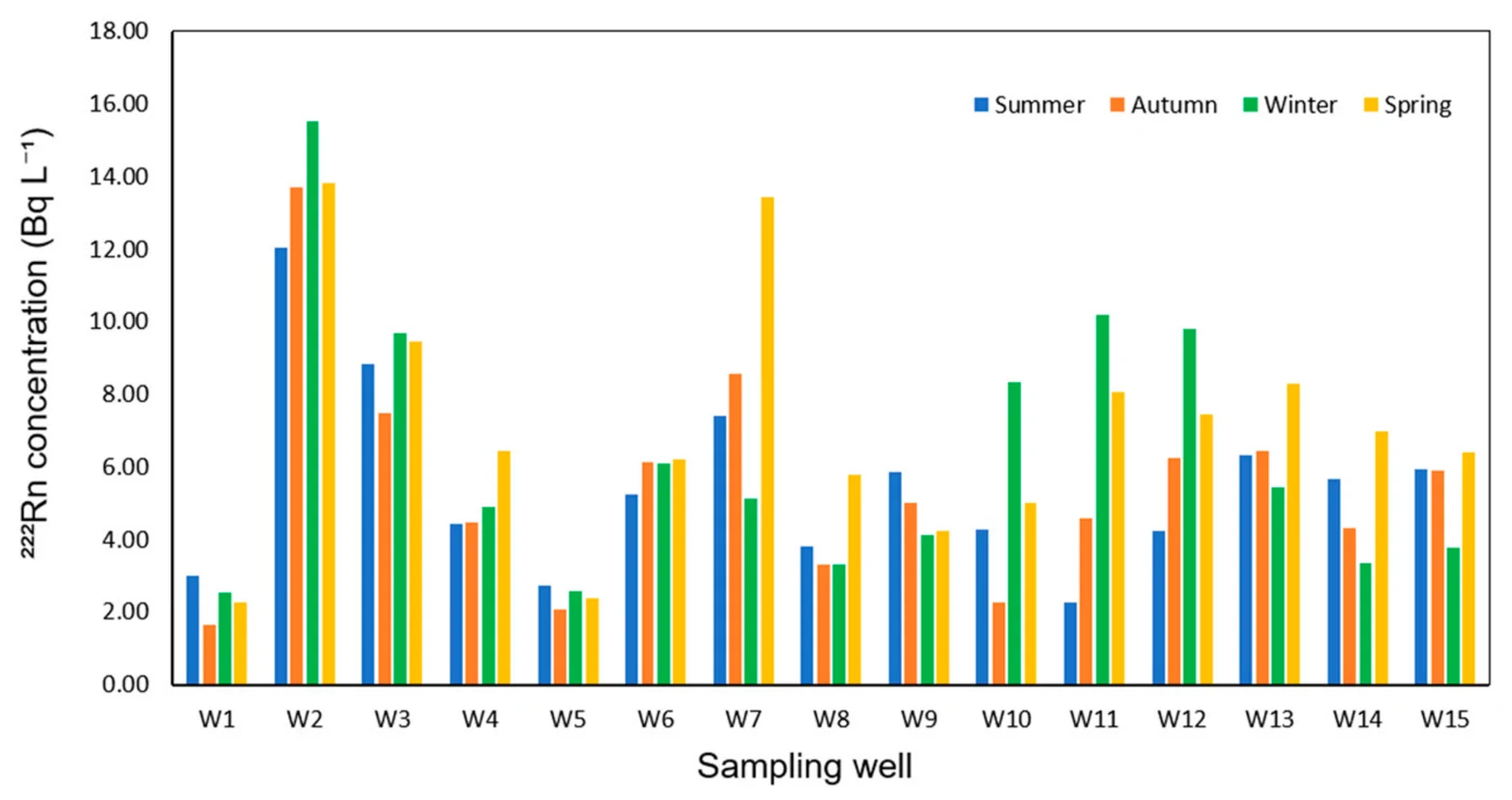
Seasonal variation in mean ^222^Rn activity concentrations in the investigated drinking water wells during the one-year monitoring period.

The seasonal mean ^222^Rn activity concentrations measured in the investigated wells are presented in Figure 7. The seasonal average ^222^Rn concentrations within the study area were calculated as 5.48 ± 2.53, 5.48 ± 3.02, 6.32 ± 3.67, and 7.08 ± 3.32 Bq L^−1^ during summer, autumn, winter, and spring, respectively. These results indicate that the average ^222^Rn concentrations in the study area tended to be relatively higher during the winter and spring seasons. The findings further reveal that ^222^Rn concentrations exhibited different seasonal patterns among the wells. While relatively elevated concentrations were observed in some wells during winter and spring, seasonal variations were found to be limited in others. Overall, the seasonal fluctuations in groundwater ^222^Rn concentrations did not exhibit a consistent trend across the study area, indicating that seasonal variability differed among the investigated wells. To account for the repeated measurements obtained from the same wells during the one-year monitoring period, a linear mixed-effects model was applied, with season treated as a fixed effect and well as a random effect. This approach accounted for the non-independence of repeated observations from the same well and distinguished within-well seasonal variation from between-well variability. The analysis identified a significant difference in groundwater ^222^Rn concentrations between spring and autumn (β = 1.604 Bq L^−1^, 95% CI: 0.674–2.534, *p* = 0.001). In contrast, no statistically significant differences were observed between summer and autumn (β = 0.000 Bq L^−1^, 95% CI: −0.930 to 0.930, *p* = 1.000) or between winter and autumn (β = 0.844 Bq L^−1^, 95% CI: −0.121 to 1.809, *p* = 0.086). The model also revealed substantial between-well variability, suggesting that differences among wells were greater than seasonal differences.

As shown in Figure 7, the differences observed among the wells persisted throughout the entire monitoring period. In particular, wells W2, W7, and W11 consistently exhibited higher ^222^Rn concentrations than the other wells across all seasons, whereas wells W1 and W5 were characterized by lower and relatively stable concentrations. Furthermore, the maximum ^222^Rn concentrations were observed at different sampling times among the investigated wells, indicating the absence of a common seasonal pattern.

Examination of the seasonal variations revealed that although elevated ^222^Rn concentrations were observed in some wells during winter and spring, this pattern was not consistent throughout the study area. For example, pronounced increases were observed during spring in wells W7 and W13, whereas higher concentrations were recorded during winter in wells W11 and W12.

The average ^222^Rn concentration in the study area was highest during spring (7.08 ± 3.32 Bq L^−1^), while lower values were observed during summer and autumn. The mixed-effects analysis further showed that groundwater ^222^Rn concentrations were significantly higher in spring than in autumn (*p* = 0.001), whereas no significant differences were detected between autumn and either summer or winter. Previous studies have reported that ^222^Rn concentrations in groundwater may exhibit seasonal variability [78,79]. Similarly, seasonal fluctuations in groundwater ^222^Rn concentrations have been documented in different hydrogeological settings [25,80]. However, the present dataset does not allow the observed seasonal variations to be directly attributed to any specific environmental factor. Moreover, seasonal patterns were not uniform among the wells; higher ^222^Rn concentrations were observed during winter in some wells and during spring in others. These findings suggest that the observed variability in ^222^Rn concentrations cannot be explained solely by seasonal influences.

The study area is located within the Akşehir–Simav Fault System, providing an active tectonic setting for investigating groundwater ^222^Rn variability. However, no statistically significant relationship was observed between groundwater ^222^Rn concentrations and distance to the nearest mapped fault. Considerable spatial variability was observed among the sampled wells, with the highest mean ^222^Rn concentrations recorded in wells W2 and W7.

It is widely recognized that ^222^Rn concentrations in groundwater can be affected by aquifer characteristics, water–rock interactions, and groundwater circulation conditions. Ref. [81] reported that radon concentrations in groundwater are influenced primarily by lithological characteristics and rock–water interactions rather than by the chemical composition of the water itself. The well-specific variations observed in the present study may likewise be associated with similar natural processes.

In the literature, health risks associated with groundwater radon are generally evaluated using data obtained from single-period or short-term measurements. In contrast, the present study is based on the average of measurements conducted at regular intervals over a one-year period and therefore provides a more comprehensive assessment that accounts for the seasonal variability of radon concentrations. This approach may contribute to a more reliable evaluation of radiological risks associated with drinking water consumption.

## 4. Conclusions

In this study, ^222^Rn activity concentrations were monitored at biweekly intervals over a one-year period in 15 drinking water wells located within the Sultandağı Fault Zone in Afyonkarahisar, Türkiye, and the associated radiological risks were evaluated. The results showed that ^222^Rn concentrations in groundwater ranged from 0.05 to 26.64 Bq L^−1^, with an overall annual mean concentration of 6.12 ± 2.79 Bq L^−1^ for all investigated wells. The highest annual mean concentration was recorded in well W2 (13.59 ± 5.86 Bq L^−1^), whereas the lowest value was observed in well W1 (2.37 ± 2.02 Bq L^−1^).

All measured average radon concentrations were below the reference level of 100 Bq L^−1^ recommended by the World Health Organization (WHO) for drinking water [43]. Furthermore, only one of the fifteen investigated wells (W2) exhibited an annual average radon concentration exceeding the 11 Bq L^−1^ limit recommended by the United States Environmental Protection Agency [42]. These findings indicate that the measured radon concentrations were generally below the international reference levels used for comparison in this study.

Seasonal evaluation revealed average ^222^Rn concentrations of 5.48 ± 2.53, 5.48 ± 3.02, 6.32 ± 3.67, and 7.08 ± 3.32 Bq L^−1^ during summer, autumn, winter, and spring, respectively. Although relatively higher concentrations were observed during winter and spring, seasonal variations did not exhibit a uniform pattern across all wells. Nevertheless, the mixed-effects analysis demonstrated significantly higher groundwater ^222^Rn concentrations in spring than in autumn over the one-year monitoring period.

Correlation analysis revealed a moderate and statistically significant positive relationship between ^222^Rn concentration and electrical conductivity (EC) (ρ = 0.529, *p* = 0.043), whereas no significant correlations were observed with well depth, pH, or water temperature.

The annual effective dose (AED) values associated with groundwater consumption ranged from 13.90 to 79.86 μSv y^−1^ for infants, 5.09 to 29.27 μSv y^−1^ for children, and 6.04 to 34.72 μSv y^−1^ for adults. The corresponding mean AED values were calculated as 35.94, 13.17, and 15.63 μSv y^−1^, respectively. Since all AED values remained below the reference levels of 100 μSv y^−1^ recommended by WHO and 290 μSv y^−1^ suggested by UNSCEAR, relatively low estimated ingestion doses were obtained under the assumptions applied in this study [1,54].

The excess lifetime cancer risk (ELCR) values ranged from 0.49 × 10^−4^ to 2.80 × 10^−4^ for infants, 0.18 × 10^−4^ to 1.02 × 10^−4^ for children, and 0.21 × 10^−4^ to 1.22 × 10^−4^ for adults. The corresponding mean ELCR values were 1.26 × 10^−4^, 0.46 × 10^−4^, and 0.55 × 10^−4^, respectively. The highest risk values were observed in well W2, highlighting the importance of elevated radon concentrations in radiological risk assessments.

Overall, the measured ^222^Rn concentrations in the investigated drinking-water wells did not indicate elevated radiological exposure from drinking-water ingestion under the assumptions used for dose assessment. Nevertheless, regular monitoring of ^222^Rn concentrations and periodic assessments of the associated radiological risk are recommended for groundwater resources used for drinking purposes, particularly for wells exhibiting relatively elevated radon concentrations and in regions characterized by active tectonic features. This study represents one of the few long-term ^222^Rn monitoring studies in drinking-water wells in Türkiye, based on biweekly measurements conducted over a one-year period in 15 wells. Through biweekly measurements conducted over a one-year period, seasonal variations in ^222^Rn concentrations and the associated radiological risk parameters were comprehensively evaluated. Compared with studies based on single-period measurements, the present work offers a more reliable assessment of radiological risks and is expected to contribute valuable information for future investigations on environmental radioactivity, hydrogeology, and groundwater quality.

## Figures and Tables

**Table 1:** Geographic locations and selected hydrogeological and physicochemical characteristics of the drinking-water wells sampled in the Sultandağı Fault Zone, Türkiye, including their distances to the nearest mapped fault.

Well ID	Location Name	Lat. (°N)	Long. (°E)	Alt. (m)	Depth (m)	pH	EC (µS cm^−1^)	Water Temp. (°C)	Fault Dist. (km)
W1	Ali İhsan Paşa	38.7110	30.6055	1008	117	7.44	654	19.5	5.4
W2	Salar	38.7141	30.6258	1008	100	6.65	1540	21	4.75
W3	Sülümenli	38.6759	30.7372	1000	45	7.11	1074	14.9	7.1
W4	Heybeli	38.6458	30.8342	1001	280	6.91	377	43	0.17
W5	Maltepe	38.6070	30.9098	1005	ND	7.33	1032	20	0.13
W6	Kadıköy	38.6373	30.9185	988	50	7.9	2010	15.3	3.27
W7	Çobanlar 1	38.6402	30.8687	976	600	7.08	1534	27.1	2.9
W8	Çobanlar 2	38.6412	30.8686	982	700	7.55	226	20	2.97
W9	Cumhuriyet	38.6020	30.9386	998	85	8.7	342	16.8	0.07
W10	Ali Kaleli	38.5979	30.9882	988	120	8.23	424	15.3	0.97
W11	Çay	38.5971	31.0415	1023	160	8.15	704	12	1.09
W12	Çayırpınar	38.5900	31.0951	996	100	7.65	722	16.2	1.59
W13	Kocaoğuz	38.6093	31.1623	983	80	8.3	357	14	4.62
W14	Yakasenek	38.5799	31.2251	991	200	8.1	422	15.3	5.72
W15	Dereçine	38.5012	31.2680	1015	10	7.95	500	18.8	1.56

ND = no data available.

**Table 2:** Minimum, maximum and annual mean (±SD) ^222^Rn activity concentrations measured in drinking-water wells during the one-year monitoring period, as well as the corresponding age-dependent annual effective doses (AEDs) and excess lifetime cancer risks (ELCRs).

Well ID	^222^Rn Concentrations Bq L^−1^			Annual Effective Doses (μSv y^−1^)			ELCR (×10^−4^)		
	Min	Max	Mean ± SD	Infants	Children	Adults	Infants	Children	Adults
W1	0.39	11.20	2.37 ± 2.02	13.90	5.09	6.04	0.49	0.18	0.21
W2	0.16	26.64	13.59 ± 5.86	79.86	29.27	34.72	2.80	1.02	1.22
W3	1.01	15.52	8.82 ± 3.11	51.83	18.99	22.53	1.81	0.66	0.79
W4	0.85	12.77	5.06 ± 1.92	29.75	10.90	12.93	1.04	0.38	0.45
W5	0.5	6.77	2.45 ± 1.08	14.37	5.27	6.25	0.50	0.18	0.22
W6	2.12	8.28	5.97 ± 1.64	35.09	12.86	15.26	1.23	0.45	0.53
W7	0.13	15.24	8.96 ± 5.89	52.65	19.29	22.89	1.84	0.68	0.80
W8	0.16	7.43	4.23 ± 2.72	24.86	9.11	10.81	0.87	0.32	0.38
W9	0.62	12.11	4.84 ± 2.96	28.44	10.42	12.37	1.00	0.36	0.43
W10	0.55	12.76	4.82 ± 3.82	28.32	10.38	12.31	0.99	0.36	0.43
W11	0.1	13.28	6.30 ± 4.07	37.03	13.57	16.10	1.30	0.47	0.56
W12	0.78	10.85	6.96 ± 3.19	40.93	15.00	17.79	1.43	0.52	0.62
W13	0.05	11.40	6.68 ± 3.09	39.25	14.38	17.07	1.37	0.50	0.60
W14	0.81	10.35	5.14 ± 2.22	30.22	11.07	13.14	1.06	0.39	0.46
W15	2.9	12.35	5.55 ± 1.97	32.62	11.95	14.18	1.14	0.42	0.50
Mean			6.12 ± 2.79	35.94	13.17	15.63	1.26	0.46	0.55

**Table 4:** Spearman correlation coefficients between mean ^222^Rn concentrations and selected hydrogeological and structural parameters.

Parameter	Spearman’s ρ	*p*-Value
Depth	−0.273	0.345
pH	−0.239	0.39
EC	0.529	0.043
Water temperature	−0.16	0.57
Distance to nearest mapped fault (km)	0.361	0.187

## Data Availability

The raw data supporting the conclusions of this article will be made available by the author on request.
